# A magnesium-induced triplex pre-organizes the SAM-II riboswitch

**DOI:** 10.1371/journal.pcbi.1005406

**Published:** 2017-03-01

**Authors:** Susmita Roy, Heiko Lammert, Ryan L. Hayes, Bin Chen, Regan LeBlanc, T. Kwaku Dayie, José N. Onuchic, Karissa Y. Sanbonmatsu

**Affiliations:** 1 Center for Theoretical Biological Physics, Rice University, Houston, Texas, United States of America; 2 Department of Chemistry and Biochemistry, University of Maryland, College Park, Maryland, United States of America; 3 Departments of Physics and Astronomy, Chemistry, and Biosciences, Rice University, Houston, Texas, United States of America; 4 Theoretical Biology and Biophysics Group, Theoretical Division, Los Alamos National Laboratory, Los Alamos, New Mexico, United States of America; 5 New Mexico Consortium, Los Alamos, New Mexico, United States of America; University of Virginia, UNITED STATES

## Abstract

Our ^13^C- and ^1^H-chemical exchange saturation transfer (CEST) experiments previously revealed a dynamic exchange between partially closed and open conformations of the SAM-II riboswitch in the absence of ligand. Here, all-atom structure-based molecular simulations, with the electrostatic effects of Manning counter-ion condensation and explicit magnesium ions are employed to calculate the folding free energy landscape of the SAM-II riboswitch. We use this analysis to predict that magnesium ions remodel the landscape, shifting the equilibrium away from the extended, partially unfolded state towards a compact, pre-organized conformation that resembles the ligand-bound state. Our CEST and SAXS experiments, at different magnesium ion concentrations, quantitatively confirm our simulation results, demonstrating that magnesium ions induce collapse and pre-organization. Agreement between theory and experiment bolsters microscopic interpretation of our simulations, which shows that triplex formation between helix P2b and loop L1 is highly sensitive to magnesium and plays a key role in pre-organization. Pre-organization of the SAM-II riboswitch allows rapid detection of ligand with high selectivity, which is important for biological function.

## Author summary

The presence of positively charged metal ions is essential to maintain the structural fold and function of RNA. Among different metal ions, magnesium is particularly important for the stability of RNA because it can efficiently support a close assembly of negatively charged phosphate groups in an RNA fold. The SAM-II riboswitch is an example of a classical pseudoknot fold, which binds S-adenosyl methionine, stabilizing an alternate folded form to inhibit gene expression. In our early ^13^C- and ^1^H-chemical exchange saturation transfer (CEST) experiments, we found a conformational transition between a minor, partially closed and a major, open state conformation in the absence of ligand. Our CEST experiments at different magnesium concentrations now suggest that magnesium ions can induce a conformational pre-organization in the apo SAM-II riboswitch, which is expected to facilitate ligand binding. To understand the microscopic details of this magnesium-induced transition, we perform all-atom structure-based molecular simulations including electrostatics and explicit magnesium ions. Our free energy calculations reveal that the partially closed pre-organized state is further stabilized with increasing magnesium concentration. This is in excellent agreement with our ^13^C-CEST profile, SAXS, and size-exclusion chromatographic data, and with recent single molecule FRET experiments. Our results suggest that a sufficiently high concentration of magnesium is essential to pre-organize the apo SAM-II riboswitch.

## Introduction

Non-coding RNAs are currently thought to account for over 75% of the human genome [1]. In bacteria, non-coding RNAs play important roles in gene regulation. One such class of RNAs, riboswitches, regulates metabolite production. Here, a single RNA sequence folds into one of two or more mutually exclusive folds depending on the metabolite concentration [2,3]. In some cases, such as the S-adenosylmethionine-I (SAM-I) riboswitch, the RNA contains a transcriptional terminator that forms when ligand is present, in effect silencing genes important for ligand production [4–6]. When the ligand is not present, the terminator does not form, allowing gene expression, and therefore ligand production, to continue efficiently. In other cases, such as the SAM-II riboswitch, ligand binding may lead to sequestration of the Shine/Dalgarno sequence, likely blocking ribosome binding and, as a consequence, protein synthesis [7]. While these examples of ligand-dependent secondary structure switches have been known for some time, a detailed thermodynamic understanding at the atomistic level, including the indispensable effect of the RNA’s ion-atmosphere, has not been achieved. In recent years, riboswitches have become canonical systems for studies of diverse RNA behaviors, as they possess quintessential characteristics of many RNA systems: ligand binding, Magnesium ion (Mg^2+^) sensitivity, conformational changes, secondary structure remodeling, and regulatory functions. Chemical footprinting, NMR, small-angle X-ray scattering (SAXS) and single molecule FRET techniques are being exploited to elucidate the folding kinetics, thermodynamics and the magnesium ion sensitivity in RNA systems such as the TPP riboswitch [8], glycine-dependent riboswitches [9], different variants of P4-P6 RNA [10–12] and P5abc subdomain of the Tetrahymena group I intron ribozyme [13]. Other work focuses on more complex functions, such as splicing and ligand recognition and their associations with proteins or different metabolites [14,15]. Success in understanding the structural, dynamical and functional aspects of riboswitch systems requires an integrated experimental and theoretical approach. Traditional crystallographic techniques produce static snapshots of the riboswitch. SmFRET, NMR, and SAXS methods obtain kinetic information and overall distributions of conformations. Molecular simulation allows one to integrate disparate experimental data into a single coherent picture, characterizing transitions in atomistic detail and the free energy landscape with fine resolution. A large number of riboswitches have been crystallized and have also been investigated via fluorescence and single molecule techniques [16–28].

Molecular simulations have also been used to study a number of riboswitches, including but not limited to the SAM-I, SAM-II, pre-Q, and adenine riboswitches [29–32]. Some of these studies replaced the essential atmosphere of divalent Mg^2+^ ions with other, monovalent ions. Others used a repulsive Debye-Hückel interaction between the phosphate groups of the RNA. Such implicit treatments of the ions, however, neglect important near-field effects that occur inside the core of the riboswitch, where the ions are strongly coupled to the RNA.

Although the role of Mg^2+^ in stabilizing the RNA tertiary structure has long been realized [33,34], the molecular basis of ion-RNA interactions, in terms of structure and function, is not well understood. In a pioneering study, Draper and co-workers distinguished three classes of ion environments: (i) the diffuse ions, which are not restrained to any particular region, (ii) water-surrounded ions separated from the RNA by a single hydration layer, which we call outer-sphere ions, and (iii) chelated ions in the inner sphere, which form direct contacts with at least two different phosphate groups of the RNA [34,35]. While the potential role of chelated ions has been emphasized in many studies, recent work using explicit solvent molecular dynamics instead highlights a dense layer of outer-sphere Mg^2+^ ions, which are primarily responsible for anchoring the RNA structure [36]. These outer-sphere Mg^2+^ ions are only transiently bound but nonetheless strongly coupled to the RNA dynamics. Such highly correlated Mg^2+^ ions may even reside in the core region of riboswitch RNAs [36,37]. This dynamic cloud of Mg^2+^ has also been simulated in all-atom reduced models that combine Manning theory with a background of monovalent ions, represented by Debye-Hückel interactions [37,38]. In addition to these native basin simulations, studies of metabolite recognition and specificity have also been initiated using conformational ensemble sampling, again in the absence of Mg^2+^ ions [39].

While most riboswitch studies have focused on riboswitches in the 5’-UTR of mRNA that control transcription, less attention has been paid to translational control by riboswitches through ligand-dependent sequestration of the ribosome binding site (i.e., the Shine/Dalgarno sequence). The SAM-II riboswitch is one such relatively small RNA element which regulates methionine and SAM biosynthesis. A single hairpin, classic H-type pseudoknot and triplex interaction near the ligand binding site make this RNA an interesting system to study RNA control over the translation initiation process [21,40–44].

A previous single molecule fluorescence resonance energy transfer (smFRET) [21] imaging study sheds light on the dynamic nature of ligand-free SAM-II riboswitch, which becomes conformationally restrained upon ligand binding. The flexibility of such highly transient conformations is tuned to ensure a viable time scale for conformational transitions in the absence of ligand. More rapid sensing could, however, be achieved if the riboswitch adopted a binding competent conformation in the ligand-free state. Mg^2+^ ions can act as effective anchors, aiding in the preservation of the structural integrity of the RNA. The emergence of two distinct FRET configurations in the presence of 2 mM Mg^2+^ in the ligand free system suggests that Mg^2+^ has the ability to compress the RNA structure, in such a way that it might pre-organize the RNA to form a binding competent conformation. In a series of small angle X-ray scattering (SAXS) experiments, we observed an analogous signature of Mg^2+^ induced structural collapse that can facilitate subsequent ligand binding [40]. Our studies provided direct insight into the global rearrangement induced by both Mg^2+^ and ligand. The compaction of RNA by Mg^2+^ was also studied by size-exclusion chromatography (SEC): changes in the measured elution volume suggested a decrease in the particles’ hydrodynamic radius [40].

Pre-organization by Mg^2+^ has also been observed in other riboswitches [21,28,45]. In the SAM-I system, our biochemical studies have shown that addition of Mg^2+^ yields the pre-organized partially folded state. In addition, we have shown that, in the absence of Mg^2+^, the fully folded state cannot be achieved, even at high ligand concentrations [23,28,46]. The presence of both, Mg^2+^ and ligand are required for the stabilization of the fully folded ligand-bound configuration.

While previous smFRET and SAXS data revealed that ligand free RNA undergoes substantial structural changes upon variation of Mg^2+^ concentration, these structural changes often remained undetected by traditional NMR and X-ray crystallography techniques because of the transient nature and low population levels of such intermediates [47]. The newly developed Chemical Exchange Saturation Transfer (CEST) measurements are now capable of probing these sparsely and transiently populated RNA conformations. Earlier we studied NMR dynamics of the SAM-II system with this new method [47]. The data indeed confirmed that SAM-II riboswitch can access a sparsely populated but bound-like pre-organized state even in the absence of ligand [40,47].

In the present study, we performed molecular simulations to predict the effect of Mg^2+^ on the conformational landscape of the SAM-II riboswitch. We then tested these predictions with ^13^C-CEST data. Analysis of our simulations yields the free energy landscape of the SAM-II riboswitch, the effect of Mg^2+^ on this landscape and insight into the microscopic origins of these effects. More specifically, reappraisal of the ^13^C-CEST data for the ligand-free SAM-II riboswitch at different Mg^2+^ concentrations enabled us to probe the influence of Mg^2+^ on sparsely populated bound-like pre-organized states. We then revisited earlier smFRET, SAXS and SEC elution profiles and compared with our present equilibrium simulation results to integrate these data into a unified scenario of Mg^2+^-induced collapse. We calculated the free energy landscape of the SAM-II riboswitch using our recently developed all-atom structure-based model (SBM) that includes explicit Mg^2+^ ions and the effects of Manning condensation and Debye-Hückel Potassium and Chloride interactions. We specifically predict that, as Mg^2+^ concentration is increased from 0.25 mM to 2 mM, the SAM-II riboswitch collapses from an extended, partially unfolded state to a highly compact, pre-organized state, in agreement with the ^13^C-CEST studies, where we observe a shift in population towards a bound-like conformation. In addition, our simulations characterize this collapse transition in terms of the radius of gyration as a function of Mg^2+^ concentration, which is qualitatively similar to previous SAXS measurements. This agreement gives us confidence in the microscopic details of our simulations, showing that the triplex formation between helix P2b and loop L1 plays an important role in the collapse process.

## Results

**Fig 1:**
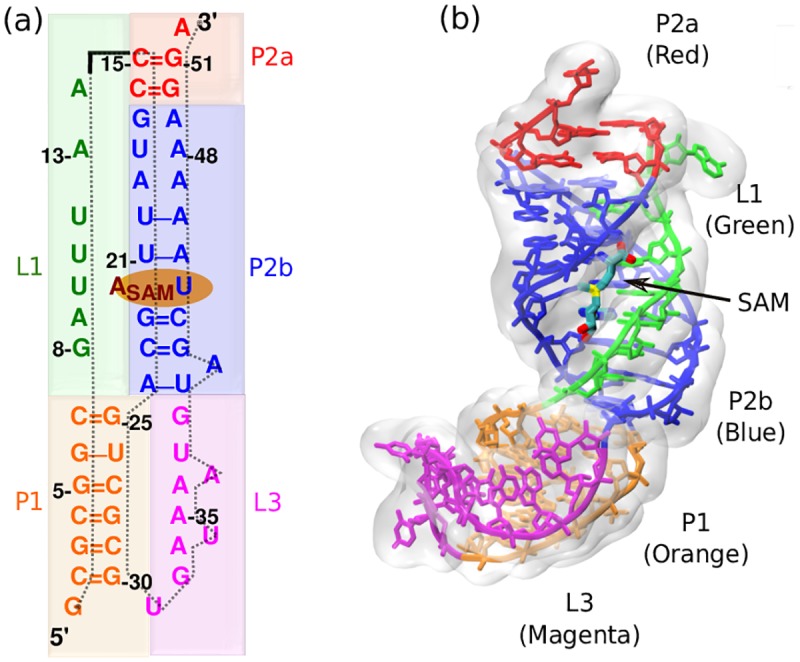
Secondary and tertiary structure of SAM-II riboswitch in ligand-bound state (pdb:2QWY). (a) Sequence-aligned secondary structure of the SAM-II riboswitch where base pair and stacking interactions are indicated. There are three helices and two strands highlighted with different colors. (P1: Orange, P2a: Red, P2b: Blue, L1: Green, L3: Magenta). (b) Tertiary structure displays triple helix between helix P2b and loop L1.

Simulations of the SAM-II riboswitch were performed with and without SAM at different Mg^2+^ concentrations. The simulation started from the crystal structure of SAM-II riboswitch (pdb accession code: 2QWY) [20]. A global view of this structure and details of the secondary structure are presented in Fig 1. As ligand-free conformations of SAM-II are still under investigation in experiments, we explore the full folding free energy landscape both in the presence of ligand (SAM) and in the absence of ligand to characterize the entire accessible conformational space. To cover the folding landscape, we employed umbrella sampling over the reaction coordinate, Q, which is the fraction of intra-molecular native contacts, present in both the free and bound states, formed by the riboswitch.

### Detailed agreement between ^13^C-CEST, smFRET, SAXS, SEC elution profile and generalized Manning model corroborates pre-organization by Mg^2+^

**Fig 2:**
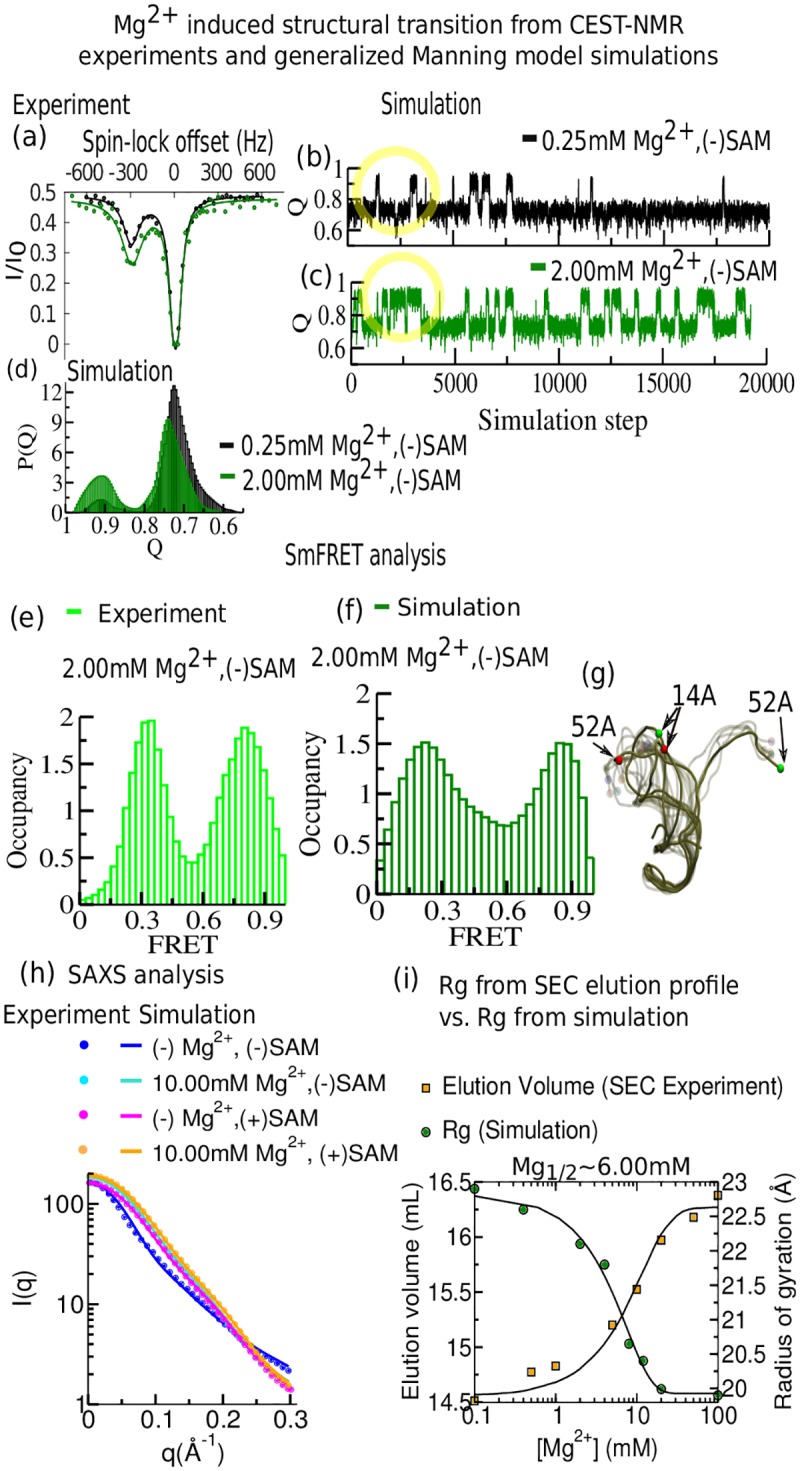
Detailed agreement between ^13^C CEST, smFRET, SAXS, and SEC elution profiles with the generalized Manning model corroborates pre-organization by Mg^2+^. (a)-(d) Comparison between CEST-NMR experiments and Manning model simulations. (a) ^13^C CEST profiles for C43-C6 of SAM-II riboswitch in the ligand-free situation in the presence of two different concentrations of Mg^2+^ (black dots: 0.25 mM, green dots: 2.0 mM) at B_1_ field of 17.5 Hz. The population distribution involving two major states was obtained by fitting each ^13^C CEST profile (solid lines) into a two-state model. (b) The fraction of native contacts (Q) dynamics extracted from simulations indicate the transition between two major states at 0.25 mM. (c) Same as (b) for 2.0 mM Mg^2+^. (d) The fraction of native contact population histograms at these two concentrations. (e)-(f) Steady state smFRET analysis from fluorescently labeled experiments and theoretical prediction from simulations. (e) Experimental smFRET occupancy histogram in the presence of 2.0 mM Mg^2+^ (adapted from ref. 22) [22]. (f) Theoretical prediction of FRET occupancy in the presence of 2.0 mM Mg^2+^. (g) Superposition of a set of different conformations at 2.0 mM Mg^2+^ depicts the prevalence of two distinct sets of ensembles. (h) Comparison of experimental scattering profiles of SAM-II collected from four different buffer conditions. Theoretical SAXS predictions are depicted by solid lines and the experimental data by dots (experimental data adapted from ref. 40). (i) Compaction of SAM-II as a function of [Mg^2+^] obtained from SEC elution profiles is consistent with a decrease in hydrodynamic radius (experimental data adapted from ref. 40), [40] in qualitative agreement with the observed folding transition in the average radius of gyration (Rg) obtained from equilibrium simulations at Mg_1/2_≈6.0 mM.

As mentioned earlier, CEST experiments are able to capture transiently populated dynamic conformations [13]. This strategy was applied to the ligand-free SAM-II riboswitch in the presence of 0.25 mM and 2 mM Mg^2+^. The ^**13**^C-CEST profiles of the labeled ribose C1’ and base C6 carbons of C43 were recorded at three different B_1_-fields (17.5, 27.9, 37.8 Hz) with a mixing time of 0.3 s at 298 K [47]. We compare the data for 0.25 mM and 2 mM Mg^2+^ concentrations at B_1_-field of 17.5 Hz (Fig 2a). The data were fit with a two-state model where the low population of the partially closed state (peaks around 300 Hz spin-lock offset) appears to increase with addition of 2 mM Mg^2+^. Consistent results were obtained from CEST profiles for other B_1_-fields of 27.9 and 37.8 Hz (Fig S1 in the S1 Text). Trajectory plots of the fraction of native contacts extracted from the generalized Manning equilibrium simulations of ligand-free SAM-II at these two concentrations clearly show the hopping between different conformations (Fig 2b). Furthermore, the dynamic transitions between the two major states (bound-like: Q≈0.9 and open: Q≈0.7) visit native-like conformations (Fig 2c) more frequently at 2 mM than at 0.25 mM Mg^2+^, as summarized in the corresponding contact histograms, P(Q) (Fig 2d). Both CEST experiments and simulation data indicate that the equilibrium shifts from the open conformations toward the native bound-like state as we increase Mg^2+^ concentration.

The signature of the existence of such Mg^2+^ induced bound-like states has also been reported in previous smFRET experiments (Fig 2e) [22]. To support our observations we have revisited some of these smFRET efficiency assessments [22] and compared them with theoretical FRET predictions obtained from our generalized Manning model simulations under similar buffer conditions. The equation used for theoretical FRET prediction is described in section S1 in the S1 Text. We tracked the dynamics of positions 14 and 52, where acceptor (cy5) and donor (cy3) fluorophore labels were placed in the smFRET experiments (Fig 2f). Both experimental and simulation FRET confirm the coexistence of two states at 2 mM Mg^2+^ (Fig 2g) [22].

Previous SAXS data corroborates well the existing smFRET observations [22,40]. The SAXS data also indicated both ligand and Mg^2+^ ions are required to effectively fold this riboswitch. To microscopically understand their mutual and stand-alone effects from the present simulations, we studied the conformational differences of this riboswitch in four extreme buffer conditions and compared our computational results with experimental SAXS data. For this comparison, we extracted multiple snapshots from several long trajectories and computed ensemble averaged SAXS profiles using the Debye formula for spherical scatterers parameterized in the FoXS web server [48,49] as described in section S2 in the S1 Text. The predicted SAXS curves here show qualitative agreement with experiments (Fig 2h) [40]. The Kratky representation of SAXS data presented in **Fig s2** in the S1 Text shows a pronounced peak, indicating the emergence of more extended conformations with decreasing Mg^2+^ concentrations. We note that capturing the entire conformational heterogeneity of an extended state is computationally challenging. This mostly applies for the extreme case where neither ligand nor Mg^2+^ is present. In this case, the correlation between theoretical and experimental SAXS profiles leaves room for improvement. Values for chi-square reflect that and are shown in **Table S1** in the S1 Text. These analyses indeed suggest the potential impact of both, ligand and Mg^2+^, stabilizing the closed conformations, which we characterize further below with contact data to describe the pre-organization and the ligand-organized closing.

A significant Mg^2+^ induced collapse transition, as indicated by the SAXS data, has been followed over a wide concentration range (up to 100 mM) of Mg^2+^ in SEC elution volume profile (Fig 2i). Here RNA elutes after longer retention times with increasing Mg^2+^ concentration ([Mg^2+^]) in the mobile phase [40]. Bigger elution volume signifies decreasing hydrodynamic radius of a monomeric RNA molecule. The folding transitions, both from experimental elution volume data and from average Rg measured from the equilibrium simulation analysis as functions of [Mg^2+^], follow sigmoid curves with transition midpoint, Mg_1/2_ at 6 mM (Fig 2i).

### Mg^2+^ remodels the free energy landscape, favoring pre-organized states

**Fig 3:**
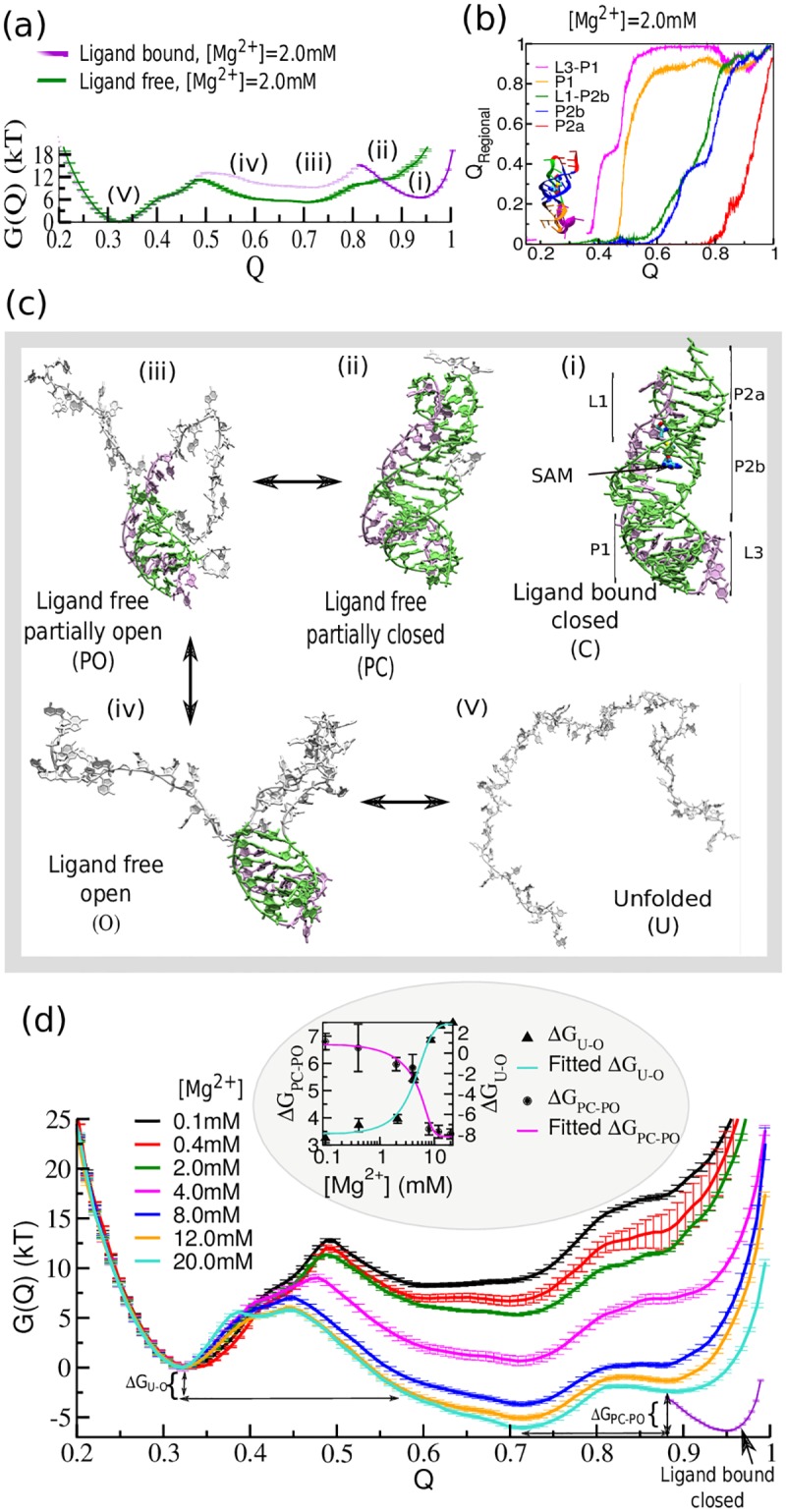
Magnesium dependence of the SAM-II riboswitch free energy landscape demonstrates Mg^2+^ pre-organization of bound-like state. (a) The free energy landscape as a function of the fraction of native contacts (Q) of SAM-II near physiological Mg^2+^ concentration ([Mg^2+^] = 2.0 mM). The system explores three distinct barrier-separated minima on the free energy landscape. (b) The order of secondary structure formation as a function of the fraction of all native contacts (Q) as measured by the fraction of non-local regional contacts (Q_Regional_). The transition displays high cooperativity. (c) Representative structures. The representative structure corresponding to each region of the energy landscape is designated as follows: (i) ligand-bound closed (C), (ii) ligand-free partially closed (PC) including triplex interactions, (iii) ligand-free partially open (PO), (iv) ligand-free open (O), and (v) the unfolded (U) state. Green/mauve, bases in native conformations. The ligand-bound closed minimum has been characterized from the free energy profile of the folding transition of SAM-bound RNA which indicates the ligand-free partially closed conformation has a substantial resemblance with the ligand-bound closed state. (d) Mg^2+^ concentration dependence of the folding transition of apo-SAM-II riboswitch. Average free energy profiles of folding transition at different [Mg^2+^] show significant stability difference between the ligand-free partially closed (PC) and the partially open (PO) minima (ΔG_PC-PO_); and the unfolded (U) and the extended open (O) state minima (ΔG_U-O_). In the inset ΔG_PC-PO_ and ΔG_U-O_ are plotted together as a function of [Mg^2+^]. Both sigmoid curves follow the same transition midpoint, Mg_1/2_ ≈6.0 mM, as found in the SEC profile.

At this point, a range of experimental techniques and simulation data support the existence of pre-organized states. Here we aim to obtain a thermodynamic description of how Mg^2+^ governs the energy landscape of RNA from our model simulation study. In Fig 3a, we show the free energy landscape for the folding transition of SAM-II riboswitch in SAM-bound (in the presence of explicit ligand) and SAM-free (in the absence of ligand) conditions near the physiological concentration of Mg^2+^ ([Mg^2+^] = 2.0 mM). During this folding transition, each secondary structural segment folds sequentially illuminating the pathway of folding (Fig 3b). The free energy profile, in the presence of explicit SAM has a distinct bound-state-well, reflecting the ligand-induced stabilization of the closed conformations (designated as (i) in Fig 3c). In the apo-form of the riboswitch, the fully closed bound state does not correspond to a minimum in the landscape. At lower Q than this ligand-bound state, the free energy profile for apo-SAM-II riboswitch reveals three distinct minima. They involve: a ligand-free partially closed state (state (ii) in Fig 3c), which has a substantial overlap with the ligand-bound closed conformation. In this state, the nonlocal contacts (involving base-pairing contacts) including base-stacking contacts in P1, the P1-L3 pseudo-knot interaction, and major segments of P2b and the L1-P2b triplex interactions remain secured, while the contacts involved in Shine/Dalgarno sequence (AAAG50G51A523´), and in the part of L1-P2b are disrupted (state (ii) in Fig 3c).

Recent fluorescence and NMR spectroscopic data also indicated that C16 in P2a helix remains mostly unpaired in the absence of SAM [22]. The data also suggested that formation of the pseudoknot in the absence of SAM is highly transient in nature. Intermediate states, (iii) and (iv) in Fig 3c, although marginally separated by a small barrier, effectively belong to a broad, flat basin which involves an ensemble of partially folded open configurations. A representative unfolded structure ((v) in Fig 3c) is shown to describe the unfolded minimum. As we increase the concentration of Mg^2+^ we find enhanced stabilization of the pre-organized partially closed conformations (state (ii) in Fig 3d) relative to the open conformations. Our latest ^13^C-CEST chemical exchange data anticipated that the emergence of Mg-induced pre-organization can have immense consequences for rapid ligand recognition [47]. In the context of the simulation, Mg^2+^ induced thermodynamic stabilization is reflected by the difference in stability, ΔG_PC-PO_ between the bound-like partially closed (PC) conformation and the partially open (PO) conformation and by ΔG_U-O_ between the unfolded (U) and the open conformation (O). These two stability differences, ΔG_PC-PO_ and ΔG_U-O_ vary upon increasing [Mg^2+^] until they reach their saturation limits. Both ΔG_PC-PO_ and ΔG_U-O_ plotted as functions of [Mg^2+^], are fitted well to sigmoid curves with a Mg^2+^_1/2_ value around 6 mM (inset of Fig 3d) which again correlates well with the SEC elution volume data (Fig 2i) [40].

### Mg^2+^-induced phosphate interactions facilitate pre-organization

**Fig 4:**
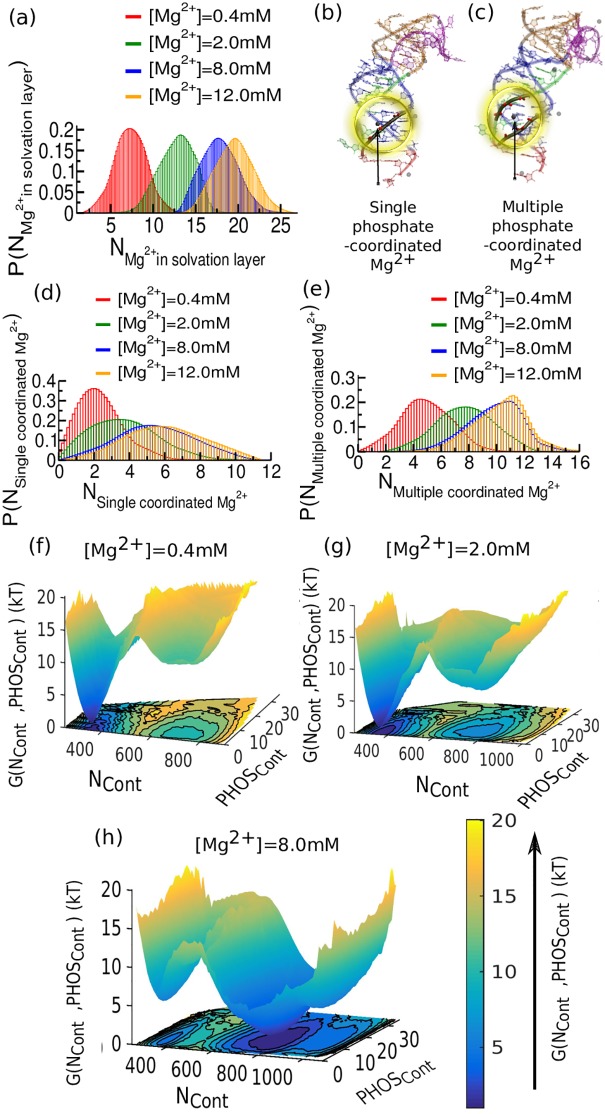
Probability distribution and free energy landscape of the SAM-II riboswitch as a function of the number of Mg^2+^ions in the ion solvation layer and Mg^2+^ mediated phosphate contacts (PHOS_Cont_). (a) The distribution of Mg^2+^ in the ion solvation layer shows gradual shift accommodating more number of Mg^2+^ with increasing Mg^2+^ concentration. Beyond 8.0 mM the first ion-solvation layer appears saturated. Subsequent additions of Mg^2+^ beyond 8.0 mM do not effectively participate in the 1st layer of solvation by ionic interaction. (b) Snapshot extracted from the simulation at buffer condition [Mg^2+^] = 0.4 mM has single phosphate coordinated Mg^2+^. (c) Snapshot extracted from the simulation at buffer condition [Mg^2+^] = 8.0 mM has multiple phosphate coordinated Mg^2+^. (d) Single phosphate coordinated Mg^2+^ as a function of Mg concentration. (e) Multiple phosphate coordinated Mg^2+^ as a function of [Mg^2+^]. The population shift of such multiple coordinated Mg^2+^ serves to connect negatively charged phosphate groups. (f) Free energy landscape as a function of N_cont_ and PHOS_Cont_ for [Mg^2+^] = 0.4 mM. (g) Same as (f) for [Mg^2+^] = 2.0 mM. (h) Same as (f) for [Mg^2+^] = 8.0 mM. Increasing [Mg^2+^] reshapes the landscape, shifting the equilibrium from a basin at lower N_cont_ to a basin at higher N_cont_.

To address the open question of how Mg^2+^ ions regulate structural collapse, we have determined the Mg^2+^ distribution in the ion-solvation layer of SAM-II, which accommodates increasing numbers of Mg^2+^ up to 8 mM Mg^2+^ content (Fig 4a). Subsequent additions of Mg^2+^ beyond 8 mM do not effectively add to the 1st layer of Mg^2+^ solvation. How we characterize the ion-solvation layer from our simulated trajectories is described in section S3 in the S1 Text (**Fig s3 in** S1 Text). We have further classified the outer sphere Mg^2+^ present in the ion-solvation layer into two categories based on their number of associated phosphate groups: (i) Single phosphate coordinating Mg^2+^ (Fig 4b), which efficiently neutralize the negative charge of the adjacent phosphate (Fig 4d), and (ii) multiple phosphate coordinating Mg^2+^ (Fig 4c). The key role in stabilizing the structure is played by such Mg^2+^ bridging multiple phosphates, which can act as glue in compact structures by holding a number of negatively charged phosphates together in close proximity.

The population shift coincident with multiple coordinated Mg^2+^ ions with increasing Mg^2+^ concentration directly supports their role in stabilizing the structure (Fig 4e). We have also investigated the thermodynamic impact of Mg^2+^-mediated phosphate contacts (PHOS_Cont_: total number of pair-wise phosphate-phosphate contacts) on the energy landscape as a function of overall folding progress, expressed by the number of native contacts (N_Cont_), as shown in Fig 4f–4h. As we increase the Mg^2+^ concentration the broad minimum that appeared around N_Cont_~800, involving partially folded open conformations, gradually becomes more stabilized. Concurrent enrichment of phosphate-phosphate contacts extends the contour of the minimum asymmetrically toward higher PHOS_Cont_. Additionally, by 8 mM [Mg^2+^], the bound-like pre-organized state grows with substantial population, stabilized again by phosphate connections (Fig 4h).

### Mg^2+^-induced triplex interactions between helix P2b and L1 loop help pre-organize the SAM-II riboswitch

**Fig 5:**
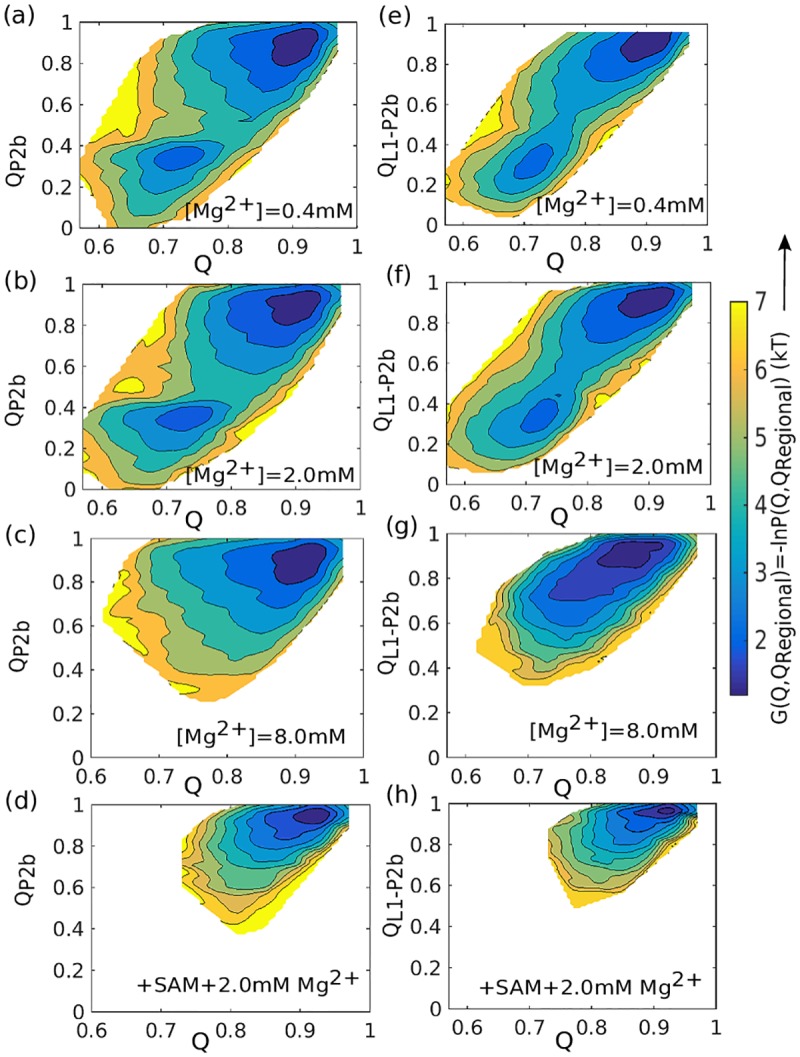
Thermodynamics of triplex formation. Free energy landscape of the SAM-II riboswitch as a function of total native contacts (x-axis) and native contacts for an individual structural element (y-axis). (a)-(c) Free energy landscapes for helix P2b formation for increasing values of [Mg^2+^], without SAM. (d) Free energy landscapes for helix P2b formation for the case of [Mg^2+^] = 2.0 mM, with SAM. (e)-(g) Free energy landscapes for triplex formation (L1-P2b contacts) for increasing values of [Mg^2+^], without SAM. (h) Free energy landscapes for triplex formation for the case of [Mg^2+^] = 2.0 mM, with SAM. (a)-(c) and (e)-(g) Collective integrity of triplex interaction (involving both P2b and P2b-L1) appears most sensitive RNA element to [Mg^2+^].

We analyzed long equilibrium trajectories of the apo- and bound-forms of SAM-II slightly below the folding temperature in order to capture the essential characteristics of the pre-organized state, and also to compare this state with the fully folded ligand bound state. We have evaluated the distribution of native contact formation in each segment of secondary structure as a function of the total Q at different Mg^2+^ concentrations. Plots show contact formation in P2b (Fig 5a–5d) and the triplex interaction between helix P2b and loop L1 (Fig 5e–5h), which are most affected by Mg^2+^ concentration. Data for the nonlocal contacts of P1, L3-P1, P2a, which appear only marginally affected by Mg^2+^ concentration, are shown in **Fig s4 in** S1 Text. The two distinct basins visible at low [Mg^2+^], for the P2b helix and L1-P2b triplex contacts correspond to the pre-organized (at higher Q) and open states (at lower Q). At increasing [Mg^2+^], the populations gradually shift towards the pre-organized state.

Around 8 mM Mg^2+^, the dominant contribution arising from this pre-organized triplex to the conformational space is evident from Fig 5c and 5g. Ligand binding also strongly favors structure formation, even at moderate [Mg^2+^], as the ligand bridges the gap between L1 strand and P2b helix, producing the fully formed triplex.

## Discussion

Motivated by our ^13^C-CEST profiles for SAM-II and their [Mg^2+^] dependence we have explored the free energy landscape of the SAM-II riboswitch using a recently developed all-atom SBM that includes explicit Mg^2+^ ions, Debye-Hückel treatment of implicit KCl interactions, and the effects of Manning condensation to accurately account for the ion atmosphere around the RNA. Our results support a mechanism involving Mg^2+^ induced pre-organization followed by conformational selection by the ligand, SAM, as we speculated in an early study [47]. The free energy analysis validates the observations of that pre-organization, providing an atomistic and thermodynamic basis for the enhanced population of a partially collapsed, pre-organized ensemble at sufficiently high Mg^2+^ concentration in the absence of ligand. We observe three distinct sets of conformations in the folding free energy landscape of ligand-free SAM-II riboswitch: (i) an ensemble of unfolded conformations, (ii) a broad ensemble of partially folded open conformations, and (iii) an ensemble of pre-organized bound-like conformations. As we increase magnesium concentration beyond 2–4 mM, the bound-like ensemble is further stabilized, shifting the equilibrium toward the pre-organized states. All the experimental results from our ^13^C-CEST profile, recent SAXS, single molecule FRET, and size-exclusion chromatographic studies are assembled and found to be in good agreement with the present simulation results (Fig 2). At higher concentrations, Mg^2+^ stabilizes compact structures by coordinating multiple charged phosphate groups of RNA in close proximity. The experimental results, together with free energy landscapes confirm that sufficient Mg^2+^ can indeed promote stable ligand binding in the SAM-II riboswitch, and is likely the structural basis for the switching control of protein translation.

While this structural pre-organization of SAM-II can assist in rapid ligand recognition, our study suggests that a sufficiently high concentration of Mg^2+^ is necessary to capture those pre-organized states. Only when the system achieves a well-organized ion solvation layer at high [Mg^2+^], the effect of additional Mg^2+^ seems limited. This layer involves a number of Mg^2+^ ions, each coordinating with multiple phosphate groups. Mg^2+^ ions thus serve as glue to the negatively charged phosphates and facilitate the structural compaction. We note that while chelated Mg^2+^ may play an important role in other riboswitch RNAs, no specific chelated ions have been reported so far in the SAM-II system.

Our molecular simulation trajectories also allow us to pinpoint the structural basis of the effect, revealing that triplex interaction between the helix P2b and its association with the L1 strand dominate the process of pre-organization as summarized in Fig 5a–5c and 5e–5g, showing the gain of structure with increasing [Mg^2+^]. In the final step, ligand binding firmly bridges the extended gap between L1 and P2b, which seems otherwise not achievable through the addition of small, dynamic Mg^2+^ alone. But although P2b and its connection with L1 can be secured by the ligand, its presence again alone cannot fully stabilize the overall structure without addition of significant amount of Mg^2+^ (Fig 2h). These findings suggest that a sufficiently high concentration of Mg^2+^ is necessary to stabilize the pre-organized triplex and then the presence ligand promotes the native triplex formation, as summarized in Fig 5d and 5h. We note that triplexes have recently emerged as important players in gene regulation by non-coding RNAs [50–53]. Base triples also play a role in RNase P and the Diels-Alder ribozyme [54]. Heroic calculations, as such recent microsecond explicit solvent simulations of riboswitches, will also shed light on these effects, especially regarding the role of solvation [55].

Nucleic acid-ion interactions make a substantial energetic contribution in the stabilization of the native state of RNAs, including complex formation with proteins and other macromolecules [56]. The dynamics of nucleic acids are also found to be strongly influenced by the motion of their ion atmospheres. Relative to other ionic species, Mg^2+^ can efficiently support a close assembly of negatively charged phosphates by mediating favorable interactions among them. Other earth alkali metals/divalent ions (e.g. Ca^2+^) and even monovalent ions are also able to induce similar transitions, albeit at higher concentration. Our early SEC elution profiles for SAM-II show that the transition midpoint in presence of Potassium (K^+^) alone occurs only at [K]_1/2_ ≈ 25 mM. The midpoint for Calcium (Ca^2+^) is [Ca]_1/2_ ≈ 8 mM, compared to 6 mM for the Mg^2+^ ion [40]. This is a direct result of the larger charge/radius ratio of magnesium [40,57].

Thus, having these special characteristics, Mg^2+^ efficiently helps pre-organize the system and enables access to the partially collapsed states that are further stabilized by ligand binding. The general importance of Mg^2+^ for the stability of compact RNA structures supports a possibly universal role of conformational selection in ligand-binding RNAs, such as riboswitches, aptamers, and possibly protein-binding RNAs. A detailed thermodynamic understanding of the underlying landscape will indeed enable greater control of riboswitch regulation, highly sought after by researchers in synthetic biology who are currently employing riboswitches as ligand-dependent ‘knobs’ to control desired gene expression [58].

## Methods

### RNA electrostatic model

Our all-atom structure-based model (SBM) has proven successful in describing the dynamics of numerous proteins and macromolecular complexes [59–63]. To elucidate RNA free energy landscapes under the influence of Mg^2+^, models capable of quantitatively describing the ion atmosphere are needed, including ionic condensation around the negatively charged phosphate groups of RNA. Early studies have simply included electrostatic effects in SBM of RNA via repulsive Debye-Hückel interactions, thus treating all ions implicitly [29,30]. Recently, our group developed a more detailed model of RNA electrostatics and applied it within all-atom structure-based molecular dynamics simulations. Our model treats Mg^2+^ ions explicitly to account for ion-ion correlations neglected by mean-field theories [38]. The KCl buffer, which completes the experimental setup, is treated implicitly by a generalized Manning counter ion condensation model [38,64], since mean-field theories correctly assess the charge densities of monovalent K^+^ and Cl^-^ ions. Classical Manning counter-ion condensation theory was originally developed for understanding the low concentration limiting behavior of polyelectrolyte chains modeled with an infinite line of charge. Folded RNA, however, is not a line of charge. To account for the compact and irregular structures of RNAs and the effects of varying ion concentrations, we improve the Manning counter ion condensation model to handle electrostatic heterogeneity, making the condensed charge density a dynamical function of each phosphate coordinate. KCl screening is characterized by a Debye-Hückel potential. Removal of the continuum screening ions from the inaccessible volume of RNA is a substantial extension to Manning counter-ion condensation. The model has been tested against experimental measurements of excess Mg^2+^ associated with RNA, characterizing the Mg^2+^-RNA interaction free energy. This hybrid SBM has opened up new possibilities to study various structural and functional processes of RNA that are essentially controlled by ions [38]. In the present study we used this recently developed all-atom hybrid SBM to understand the conformational transition of SAM-II and the corresponding Mg^2+^ sensitivity. The energy function used in this model is given below,

$$\Phi =\Phi_{\text{SBM}}+\Phi_{\text{Mg-Size}}+\Phi_{\text{ion-effect}}$$ (1)

where, Φ_SBM_ is the all-atom SBM potential ensuring a global minimum in the landscape for the native state of RNA. The SBM potential is composed of two general types of interactions:

$$\Phi_{\text{SBM}}=\Phi_{\text{local}}+\Phi_{\text{non-local}}$$ (2)

where, Φ_local_ characterizes the local interactions that encode covalent bonds and torsional angles, maintaining the correct local geometry and chirality. Φ_non-local_ comprises two non-local contributions: (i) an attractive term that is applied specifically to all tertiary interactions determined from the native structure, (ii) the general repulsive interactions, that describe the excluded volume by symmetric hard potentials (to avoid any unwanted chain crossing). Φ_Mg-Size_ adds the excluded volume interactions involving the explicit Mg^2+^ ions, regulating RNA-Mg^2+^ and Mg^2+^-Mg^2+^ interactions. Φ_ion-effect_ accounts for all interactions between charges in the system which consist of the fixed charge distribution of the RNA and the dynamic contribution from the ions. Mg^2+^ and phosphate charges interact via a Debye-Hückel potential with a screening term that depends, in turn, on the distribution of the monovalent ions. The monovalent ions, K^+^ and Cl^-^ from the added salt, fall into two categories: screening ions and Manning condensed ions. The screening ion density is obtained using Debye-Hückel electrostatics. The density of the Manning-condensed ions is modeled as the sum of two normalized Gaussian distributions where the center of each Gaussian is located on the position of the negatively charged phosphate group. All the condensation variables along with the explicit Mg^2+^ and RNA coordinates are evolved with Langevin dynamics [38]. The mathematical formulations of all the terms and the related parameterizations are discussed in depth in section S4 in the S1 Text.

### Free energy simulations

The umbrella sampling method [65] was used to sample the conformational space of SAM-II riboswitch along the reaction coordinate, Q, which is the fraction of intra-molecular native contacts in the riboswitch. The Weighted Histogram Analysis Method [66] was then used to calculate the thermodynamic quantity, G(Q). The detail is described in section S5 in the S1 Text.

### ^13^C-CEST experiments

CEST data were collected using a pseudo-3D HSQC experiment with the B_1_ field offsets (-600 to 600 Hz) incremented in an interleaved manner with 3 references (no CEST period) [47]. A total of 1024x16 complex points were recorded [40] with 32 transients with a recovery delay of 1.5 s for a total experimental time of approximately 12 hr for each spin-lock field. A CEST saturation period of 100 ms was used for the base and 200 ms for ribose. The pulse program used was an adaptation of a previously published one without the need for selective pulses [47].

### Analysis of relaxation parameters

We used a two-state model to fit each of the three profiles of the selectively labeled carbon (ribose C1’ and base C6) and quantitatively extracted the carbon chemical shift (Δω), the exchange rate, and the population of the minor state based on the Bloch−McConnell 7x7 matrix [47]. The CEST data was plotted as I(t)/I(0) versus spin-lock offset (Hz) and was fit by numerically solving the matrix exponential for the CEST spin-lock period based on this 7x7 two-state Bloch-McConnell equation as described earlier [47,67].

### FRET

In experiments, Fluorescence Resonance Energy Transfer (FRET) efficiency is the quantum yield of the energy transfer where a donor chromophore from its excited electronic state may transfer its energy to an acceptor chromophore through a non-radiative dipole-dipole coupling. The FRET efficiency varies with the separation between donor and acceptor fluorophores following the Fӧrster relation. For theoretical FRET predictions we use the Fӧrster relation where the value of Fӧrster radius is taken as 53Å [68]. We described it in detail in section S1 in the S1 Text.

### SAXS analysis

In SAXS experiments, the scattering intensity is measured from the electron density difference between the purified sample and that of the solvent/buffer. FoXs is a method that uses the Debye formula by which a theoretical scattering profile of a structure can be computed [48,49]. The detail is discussed in section S2 in the S1 Text.

## Supporting information

S1 Text Supporting figures, table and text. (PDF)
