# Supplementary material for: A magnesium-induced triplex pre-organizes the SAM-II riboswitch
Source: PLoS Comput Biol. 2017 Mar 1;13(3):e1005406. doi: 10.1371/journal.pcbi.1005406 (PMC5352136; doi:10.1371/journal.pcbi.1005406)
Supplement: S1 Text — (PDF) [file pcbi.1005406.s001.pdf]

Supporting Information for

A magnesium-induced triplex pre-organizes the SAM-II riboswitch

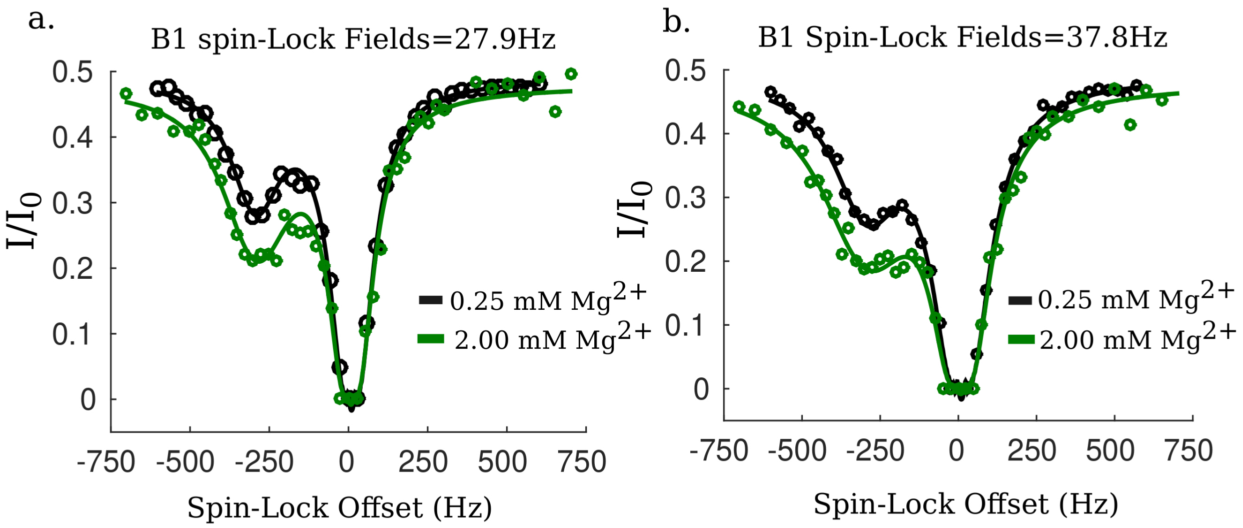

Fig S1: ^{13}C CEST profiles for C43-C6 (circle data points) of SAM-II riboswitch in ligand-free situation in the presence of two different concentrations of Mg^{2+} at B1 fields: (a) 27.9 Hz, (b) 37.8Hz. The population distribution of two major states obtained by fitting ^{13}C CEST profile (solid lines) into two states model using the Bloch-McConnell 7×7 matrix.

S1: FRET Prediction:

In experiments Fluorescence Resonance Energy Transfer (FRET) efficiency is the quantum yield of the energy transfer where a donor chromophore from its excited electronic state may transfer its energy to an acceptor chromophore through non-radiative dipole-dipole

coupling. The FRET efficiency E_{FRET} varies with the separation between donor and acceptor fluorophores (R) following the Förster relation,

$$E_{FRET} = \frac{1}{1+(R/R_0)^6} \qquad \text{(s1)}$$

where, R_0 is Förster distance at which energy transfer efficiency becomes 50%. Cyanine dyes, for instance, cy3-cy5 pairs are commonly used fluorophores in single molecule oligonucleotide studies [1]. These are often covalently linked to the 5′ and 3′ termini of DNA or RNA with a long linker. Therefore tethering the fluorophores via a reasonable linker length balances its free rotation as well as approximate coaxial separation, when dyes are in well-organized native state conformations. Steady state and time-resolved anisotropic measurements evaluated the orientation factor, (κ) where $\left(\kappa^2 = {}^2\!/_3\right)$ assuming a complete averaging over all possible configurations within the lifetime of the donor's excited state [2]. In the present work, we compared the distance between phosphates groups of the nucleoside position 14 and 52 in its closed and open conformations with that obtained from FRET experiment labeling the same position with cy5 and cy3, respectively. We determined the distance distribution along the simulation trajectory and converted into FRET efficiency following the Förster relation where R_0 =53Å [2]. In simulation we assumed the dyes' presence by adding an approximate separation ~20Å due to the presence of linkers in both ends to ensure the co-axial separation between dye-pair, preferentially in their close conformations. Thus with addition of Mg^{2+} we find a bimodal distribution in the FRET efficiency profile with the emergence of a closed inter-dye separation peaks around FRET efficiency, 0.85-0.90, both in experiment and simulation. Later on, SAXS measurement will shed more light on the Mg-induced pre-organized state.

S2. Small angle X-ray scattering (SAXS) intensity profiles using FoXS Server

In SAXS experiments, the scattering intensity is measured from the electron density difference between the purified sample and that of the solvent/buffer [3]. FoXS is a method that uses the Debye formula by which a theoretical scattering profile of a structure can be computed.

$$I(q) = \sum_i \sum_j f_i(q) f_j(q) \frac{\sin(qr_{ij})}{qr_{ij}} \quad \text{(s2)}$$

where, the scattering intensity, $I(q)$ is a function of the momentum transfer vector, $q = 4\pi \sin(\theta)/\lambda$. 2θ is the scattering angle and λ is the wavelength of the incident X-ray beam. In the Debye formulae r_{ij} is the distance between atoms i and j. The form factor, $f_j(q)$, in the model is expressed by the following relation and probes the modified solvation layer.

$$f_i(q) = f_v(q) - c_1 f_s(q) + c_2 s_i f_w(q) \quad \text{(s3)}$$

where, $f_v(q)$ is the atomic form factor in vacuo and $f_s(q)$ is that of the dummy atom. s_i is the fraction of solvent accessible surface of the atom, i and $f_w(q)$ is the form factor for water. c_1 ($0.95 \le c_1 \le 1.12$) is the scaling factor for the effective excluded volume of the atoms with a default value 1.0 and c_2 ($0 \le c_2 \le 4.0$) is for scaling the density of bulk solvent in the solvation layer. The computed profile is fitted to an input experimental SAXS profile by minimizing the χ function given by,

$$\chi = \sqrt{\frac{1}{M} \sum_{i=1}^{M} \left(\frac{I_{\text{Exp}}(q_i) - cI(q_i)}{\sigma(q_i)} \right)^2} \quad \text{(s4)}$$

where, $I_{\text{Exp}}(q_i)$ is the experimental SAXS intensity with experimental error $\sigma(q_i)$ over M number of points and $I(q_i)$ is the predicted theoretical SAXS estimated given the simulated structural snapshots in pdb format, and c is a scaling factor that minimizes χ. We have evaluated each theoretical SAXS profile from a large ensemble of structures extracted from our equilibrium simulation trajectories and computed the average SAXS profile for each condition. Here, our theoretical Rg calculations are not from Guinier approximation as experimental SAXS fitting does. Rg is determined averaging over our several equilibrium trajectories. We showed the comparison of experimental scattering profiles of SAM-II collected in different conditions in **Fig S2**. Here we add the comparison of the SAXS data in Kratky representation. To determine the statistical similarity between experimental SAXS profiles and theoretical SAXS profiles we have used reduced chi-square analysis [4]. Values for the χ^2-agreement and a comparison between Rg evaluation obtained from those experimental SAXS profiles and Manning model simulations at different buffer conditions are depicted in **Table S1**.

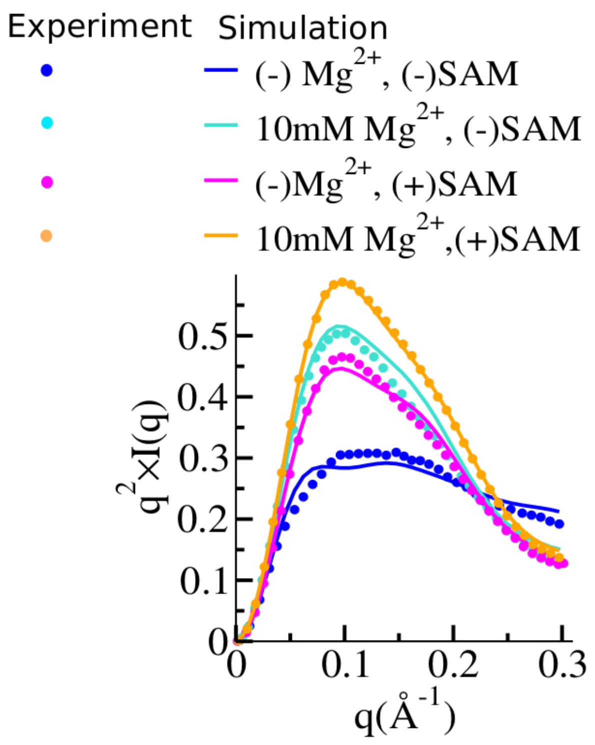

Fig S2. SAXS data in Kratky representation for SAM-II riboswitch in different buffer conditions. Theoretical SAXS predictions are represented by solid lines and the experimental data are represented by dots (experimental data adapted from ref. 40 from the main text).

Table S1: Comparison between average Rg values calculated from SAXS experiments and Manning model simulations and χ^2-values between experimental and theoretical SAXS measurements.

[Mg^{2+}]	*SAM*	*Rg, Manning Model Simulation*	*Rg, SAXS Experiment*	*SAXS χ2*
**-**	-	28.3 Å	31.7 Å	1.79
**10 mM**	-	20.6 Å	21.5 Å	1.32
**-**	+	20.5 Å	20.7 Å	0.64
**10 mM**	+	19.4 Å	19.5 Å	0.22

S3: Characterization of ion-solvation layer:

We have set the boundary of first layer of the ion-solvation by evaluating the radial distribution function g(r) of Mg^{2+} around the phosphate groups of the riboswitch. In the radial distribution curve after the maxima appeared around 0.5nm, there is an initial rapid fall with a steep slope as we increase the distance (r). Around 1.4nm this rapid fall slows down significantly and a shoulder appears as a signature of an interface [5]. Thus 1.4nm cut-off is considered as a boundary wall between first layer and second layer of ion-solvation. In **Fig S3**, we have highlighted this boundary. We tracked the coordination number of Mg^{2+} interacting with phosphate groups in this ion-solvation layer. As this coordination is made by the direct interaction between a P atom and an Mg^{2+} ion, we have chosen a rather stringent cutoff, 0.7nm, which is also the Bjerrum length and beyond which such interactions are usually screened.

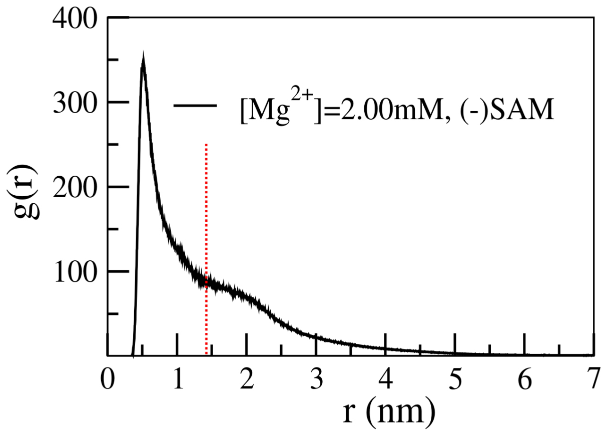

Fig S3. Radial distribution function of Mg^{2+} around the phosphate groups of SAM-II riboswitch. The boundary of 1st ion solvation layer in g(r) is highlighted by red dotted line.

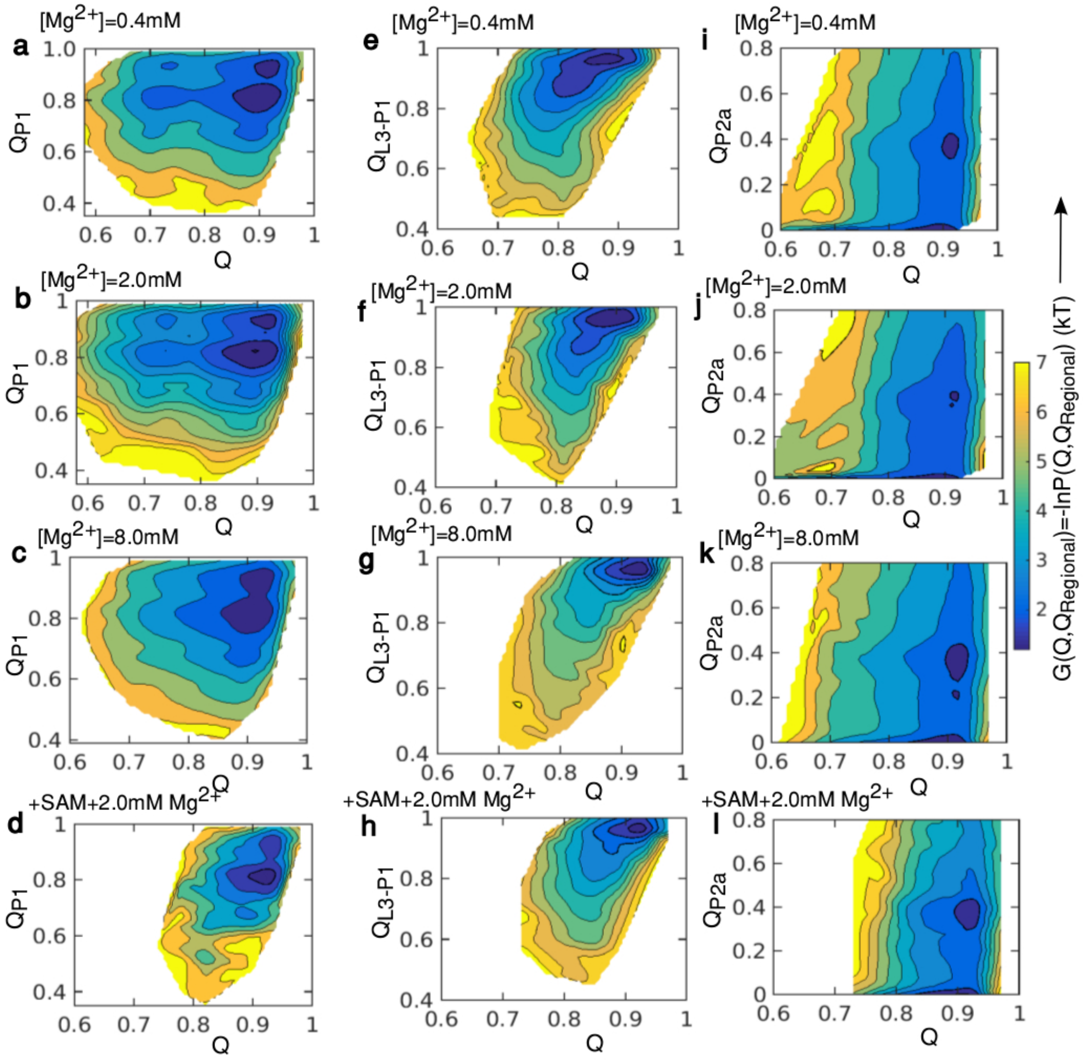

Fig S4. Integrity of secondary structural segments (those are less sensitive to Mg^{2+}) at various Mg^{2+} concentrations and in presence and absence of ligand. Population-based free energy contours are plotted as a function of segment-specific non-local native contacts ($Q_{Regional}$) and global native contacts (Q) (a)-(d) P1 helix at different buffer conditions: (a) $[Mg^{2+}]$=0.4mM, (b) $[Mg^{2+}]$=2.0mM, (c) $[Mg^{2+}]$=8.0mM, (d) +SAM+$[Mg^{2+}]$=2.0mM. (e)-(h) L3-P1 non-local contacts at: (e) $[Mg^{2+}]$=0.4mM, (f) $[Mg^{2+}]$=2.0mM, (g) $[Mg^{2+}]$=8.0mM, (h) +SAM+$[Mg^{2+}]$=2.0mM. (i)-(l) For a small segment, P2a non-local contacts at: (i) $[Mg^{2+}]$=0.4mM, (j) $[Mg^{2+}]$=2.0mM, (k) $[Mg^{2+}]$=8.0mM, (l)+SAM+$[Mg^{2+}]$=2.0mM. P1 and L3-P1 contacts in apo-SAM-II are less flexible than P2b and L1-P2b contacts irrespective of the concentration range of $[Mg^{2+}]$ or conditions chosen here in the equilibrium condition. Non-local contacts of P2a helix, however, reflect its fluctuating behavior at the terminal.

S4. To help the reader this section summarizes the model used in this work but developed in an early work by our group. The detailed description of this model can be found in **ref 7**.

A. The comprehensive form of Φ_{SBM}:

As we mentioned in the main text, Φ_{SBM} comprises two terms: Φ_{local} and $\Phi_{non-local}$. The comprehensive forms of these two terms are, respectively,

$$\Phi_{local} = \sum_{i}^{bonds} \frac{\varepsilon_r}{2}\left(r - r_{Oi}\right)^2 + \sum_{i}^{angles} \frac{\varepsilon_\theta}{2}\left(\theta_i - \theta_{Oi}\right)^2 + \sum_{i}^{\substack{proper\\dihedral}} \varepsilon_\phi F_D\left(\phi_i - \phi_{Oi}\right) + \sum_{i}^{\substack{improper\\orplanner}} \frac{\varepsilon_\chi}{2}\left(\chi_i - \chi_{Oi}\right)^2 \quad \text{(s5)}$$

$$\Phi_{Non-local} = \sum_{ij}^{contacts} \varepsilon_c \left(\left(\frac{\sigma_{ij}}{r_{ij}}\right)^{12} - 2\left(\frac{\sigma_{ij}}{r_{ij}}\right)^{6} \right) + \sum_{ij}^{\substack{RNA-RNA\\non-contacts}} \varepsilon_{NC} \left(\frac{\sigma_{NC}}{r_{ij}}\right)^{12} \quad \text{(s6)}$$

where the dihedral potential is,

$$F_D\left(\phi\right) = -\cos\left(\phi\right) - \frac{1}{2}\cos\left(3\phi\right) \quad \text{(s7)}$$

The local potential encodes the bonded geometry around each represented (non-hydrogen) atom maintaining its local geometry with bond length to its nearest neighbor, r, three body angle, θ, and four body dihedral, ϕ. The non-local interactions involve the contacts between atoms i and j with distance r_{ij} and are described by a 6-12 Lennard Jones' potential. Note that all terms in the SBM are formulated relative to values from the native structure with only one exception, the non-local excluded volume term. All the geometric parameters $(r_{Oi}, \theta_{Oi}, \chi_{Oi}, \phi_{Oi}, \sigma_{Oi})$ are set to their values from the crystal structure. The shadow method was used to obtain the native contact map [6]. Excluded-volume parameters are $\sigma_{NC} = 1.7$ Å and $\epsilon_{NC} = \epsilon_R$, where $\epsilon_R = 1.5\ k_B T$, where T is the temperature [7].

In the all-atom structure-based model the parameters are scaled in reduced units.[7] The scaling follows the definition given below:

$$\tau_R = 2 \text{ ps} \qquad \text{(s8)}$$
$$\mu_R = 15 \text{ amu} \qquad \text{(s9)}$$
$$\varepsilon_R = 3.75 \text{ kJ/mol} \qquad \text{(s10)}$$
$$T_R = 3.75 \text{ K} \qquad \text{(s11)}$$

In reduced unit, the room temperature 300K corresponds to $80T_R$. The thermodynamic sampling in our simulations was performed at high temperature to obtain a good sampling for both the ligand-bound and the ligand-free state of SAM-II riboswitch. At the same time we thus ensured that ligand association in the ligand-bound state preserved. We performed the simulations using stochastic dynamics in the under-damped limit (drag coefficient used $1.0\tau_R^{-1}$ (0.5ps^{-1})) with a time step of$0.001\tau_R^{-1}$. We followed similar energetic parameter as we have used in our early work [7]. They are:

$$\varepsilon_r = 20000\varepsilon_R / nm^2 \qquad \text{(s12)}$$
$$\varepsilon_\theta = 80\varepsilon_R / rad^2 \qquad \text{(s13)}$$
$$\varepsilon_{\chi(planar)} = 40\varepsilon_R / rad^2 \qquad \text{(s14)}$$
$$\varepsilon_{\chi(improper)} = 10\varepsilon_R / rad^2 \qquad \text{(s15)}$$

The contact and dihedral energies are balanced depending on the total number$(N)$of heavy atoms in the system. They are defined as,

$$\sum_{i}^{conatcts} \varepsilon_C = \frac{2}{3} N \varepsilon_R \qquad \text{(s16)}$$

$$\sum_{i}^{\substack{proper\\dihedral}} \varepsilon_\phi = \frac{1}{3} N \varepsilon_R \qquad \text{(s17)}$$

B. Mg^{2+} excluded volume in the potential

$\Phi_{Mg-Size}$ accounts for the excluded volume of explicit Mg^{2+} ions and is expressed as follows:

$$\Phi_{Mg-Size} = \sum_{ij}^{Mg-RNA} \varepsilon_{MgRNA} \left(\frac{\sigma_{MgRNA}}{r_{ij}} \right)^{12} + \sum_{ij}^{Mg-Mg} \varepsilon_{MgMg} \left(\frac{\sigma_{MgMg}}{r_{ij}} \right)^{12} \qquad \text{(s18)}$$

where the parameters are, σ_{MgRNA} = 3.4 Å and σ_{MgMg} = 5.6 Å [8].

Thus the ion-affinity to RNA is determined by the size of the ions in terms of their excluded volume, as well as by ε_{MgRNA}, where, $\varepsilon_{Mg-RNA} = \varepsilon_{MgMg} = \frac{4}{3} k_B T_R$.

As outer sphere Mg^{2+} ion coordinates six water molecules in its surrounding, and a hexa-hydrated Mg^{2+} ion has a reduced mass of 132 amu. To accelerate the sampling process, in our simulation, a decreased mass of Mg^{2+} ($1\mu_R$) was used. In our early studies the explicit Mg^{2+} ions distribution has been predicted by the measurement of excess Mg^{2+} surrounding an RNA molecule which has been compared with experimental evaluation and explicit solvent simulations data [8,9]. Our generalized Manning model now extends the scope to deal with any arbitrary RNA conformation, non-limiting KCl concentrations and ion inaccessible volume of RNA.

C. Ion condensation contribution ($\Phi_{ion-effect}$) to the potential

This extends the discussion of $\Phi_{ion-effect}$ which accounts for the overall charge density of the system, which is a sum of the fixed charge distribution of the RNA and the part contributed from the dynamic ions. Among the dynamical charges, from the added salt, monovalent cations (K^+) and anions (Cl^-) are of two types: (i) screening ions, (ii) Manning condensed ions.

The density of Manning condensed ions of type s, around each negatively charged phosphate, i is modeled as the sum of two normalized Gaussian distributions, $P(r,\sigma)$, centered at the position of the phosphate, i : (a) mixing Gaussian charge density, $n_{\mu,s}(\vec{r})$ (Gaussian size σ_μ is taken as 7Å, on the basis of Bjerrum length), which regulates the free energy of mixing, and (b) the hole Gaussian charge density, $n_{\eta,s}(\vec{r})$ where the σ_η is taken as 3.4Å (on the basis of Mg-RNA excluded volume effect). This hole charge density determines the ion accessibility near the RNA charges.

The competition between electrostatic energy and mixing entropy results in classical Manning counter-ion condensation on an infinite line of charge. We discussed in the main text that in the present case, as we are dealing with an irregular structure of RNA and intermediate salt concentration limits, classical Manning approach needs special treatments. Most importantly, the contribution from the screening ions needs to be included to generalize mixing free energy and electrostatic free energy and the hole volume charge density needs to be excluded to account for an effective charge density within the volume $\left(V_{\mu,i} - V_{\eta,i}\right)$.

Therefore, the total electrostatic free energy of any condensed ions can be expressed in terms of Debye-Huckel interactions between two Gaussian (with variances, σ_m^2 and σ_m^2) charge densities, $\phi\left(r_{ij}, \sqrt{\sigma_m^2 + \sigma_n^2}\right)$. Thus, the total electrostatic free energy considering both condensed and screening ions at intermediate salt concentration limit is,

$$G_E = \frac{1}{2}\sum_{ij}\sum_{mn} q_{m,i} q_{n,j} \phi\left(r_{ij}, \sqrt{\sigma_m^2 + \sigma_n^2}\right) + \frac{1}{2}\sum_{i}\sum_{s} z_s c_s \left(1 - \frac{z_s \Phi_\mu(\vec{r_i})}{k_B T}\right)\left(V_{\mu,i} - V_{\eta,i}\right)\Phi_\mu(\vec{r_i}) \qquad \text{(s19)}$$

$$\text{where, } \Phi_m(\vec{r_i}) = \sum_j \sum_n q_{n,j} \phi\left(\vec{r} - \vec{r}_j, \sqrt{\sigma_m^2 + \sigma_n^2}\right) \qquad \text{(s20)}$$

Here r_{ij} is the distance between particles i and j. z_s is the charge and c_s is the concentration of a specific ion, s. For particle i, the condensed charges are, $q_{\mu,i} = \sum_s Z_s \mu_{is}$ and $q_{\eta,i} = \sum_s Z_s \eta_{is}$. For point charges, at $\sigma_0 = 0\ nM$, the charge of particle i is $q_{0,i}$. The indices m and n covers the three cases, $\{0, \mu, \eta\}$, respectively, representing points charge, and mixing and hole Gaussians charges.

On the other hand, the mixing free energy is expressed as,

$$G_{Mix} = \sum_i \sum_s k_B T n_{Mix,is} \left(V_{\mu,i} - V_{\eta,i}\right) \ln\left(\frac{n_{Mix,is}}{e c_s}\right) \qquad \text{(s21)}$$

$$\text{where, } n_{Mix,is} = c_s \left(1 - \frac{z_s \Phi_\mu(\vec{r_i})}{k_B T}\right) + n_{\mu,s}(\vec{r}_i) \qquad \text{(s22)}$$

In order to enforce the effect of RNA excluded volume on the charge densities, we have applied strong harmonic constraints to the potential for restraining the hole charge density from the excluded volume of ith phosphate.

$$G_{Hole,is} = \frac{1}{2} k_{Hole} \sum_i \sum_s n_{Hole,is}^2 \qquad \text{(s23)}$$

where, $n_{Hole,is} = c_s \left(1 - \frac{z_s \Phi_\mu(\vec{r}_i)}{k_B T} \right) + n_{\mu,s}(\vec{r}_i) + n_{\eta,s}(\vec{r}_i)$ (s24)

In addition, μ_{is} and η_{is} are also weakly restrained to $n_{\mu,s}(\vec{r}_i)V_{\mu,i}$ and $n_{\eta,s}\left(\vec{r}_i\right)V_{\eta,i}$ by harmonic forces. The value of k_{Hole} is taken such that $k_{Hole} = 10^4 k_B T / P(0,\sigma_\eta)^2$. The tethering has been added in the form of a potential as,

$$G_{\text{Rest}} = \frac{1}{2} k_w \sum_i \sum_s \left(\mu_{is} - n_{\mu,s}(\vec{r}_i) V_{\mu,i} \right)^2 + \frac{1}{2} k_w \sum_i \sum_s \left(\eta_{is} - n_{\eta,s}(\vec{r}_i) V_{\eta,i} \right)^2 \qquad \text{(s25)}$$

Thus, $G_{Ion-Effect}$ (where, $G_{Ion-Effect} = G_E + G_{Mix} + G_{Hole} + G_{Rest}$), in terms of $\Phi_{Ion-Effect}$ contributes, in part, to the overall potential, Φ. Therefore, Φ essentially directs the four condensation variables for every phosphate $\left(\mu_{i+}, \mu_{i-}, \eta_{i+}, \eta_{i-}\right)$ along with the explicit Mg^{2+} and RNA coordinates to evolve with Langevin dynamics [7].

In our model, explicit Mg^{2+} has charge +2, and each phosphate group bears -1 charge. The details of these ionic contributions, formulation and all their related parameters can be obtained from our earlier work [7]. The model restricts condensation within an effective volume. The volume is bounded by two cutoff parameters: σ_{MgRNA} to characterize the closest approach of condensed ions to RNA, and the Bjerrum length (7Å), which confines a condensed ion to

balance the effective electrostatic and the thermal energy. The model efficiently makes KCl condensation a dynamical quantity dependent on atomic coordinates and calculated from physical principles. Since the potential is now formulated in terms of atomic coordinates, and since the KCl condensation and the phosphate-phosphate repulsion are intrinsically included, the condensation effect responds to conformational changes beyond the native basin.

S5: Umbrella sampling and unbiased equilibrium simulation detail

In a system, if a state "A" described by its reaction coordinate, X_A (which in our case is the fraction of native contact) is separated from another state "B" described by its reaction coordinate, X_B, by a finite barrier, the free energy of transition from A to B can be expressed as,

$$F(X_B) - F(X_A) = -k_B T \ln \frac{\langle P(X_B) \rangle}{\langle P(X_A) \rangle} \qquad \text{(s26)}$$

where, $\langle P(X_B) \rangle$ is the probability to find the system in the state B at the reaction coordinate, Q_B. Same holds for $\langle P(X_A) \rangle$. If these two states are separated by a high free energy barrier, umbrella sampling technique is then a useful tool which is often employed to efficiently sample the near barrier region (to surmount the barrier) along the reaction coordinate with the help of an artificial biasing umbrella potential, V. The form of V over the reaction coordinate, R is expressed as, $V = 1/2k(X - X_0)^2$, where values for X_0 are chosen between 0 and 1, to span the reaction coordinate from fully unfolded to fully folded where k is harmonic force constant.

Despite, the fraction of native contact, Q is found be a good reaction coordinate for biomolecular sampling process, the contact counting potential (V) is a step function along the reaction

coordinate. But an umbrella potential must be differentiable. Therefore, we use a continuous function of contact potential which is given as,

$$V_Q = \sum_{ij}^{contacts} \frac{1}{2}\left(1-\tanh\left(\alpha ij\right)\right) \qquad \text{(s27)}$$

where, $\alpha_{ij} = \gamma\left(r_{ij} - 1.5\mu_{ij}\right)$. r_{ij} is the separation between atoms *i* and *j,* and μ_{ij} is the same in the native state. The value of V_Q can be used as argument for the harmonic umbrella constraint during sampling.

To ensure equilibration we generated a series of initial structures at the desired Q values and then reinitialized Mg^{2+} distributions for each umbrella window. To check the consistency of the results we performed four independent repeats of the same procedure for each concentration of Mg^{2+}. Temperature was chosen such that both folded and extended states were accessible at physiological Mg^{2+} concentration ([Mg^{2+}]≈2mM). To make different Mg^{2+} composition we have set up a large cubic box of length 75nm. The number of Mg^{2+} ions included in that box determines the overall concentration of Mg^{2+}. Periodic boundary conditions were applied. Simulations of 10 million steps each were performed for a total of 50 windows along the reaction coordinate, ensuring their substantial overlap in the conformational space. As the nucleic acid chains showed highly cooperative dynamics, extended time simulations were required for good sampling of relevant conformations. Four sets of simulations were performed for SAM-free and SAM-bound states at each Mg^{2+}concentration.The error bars for the present study show the standard deviation of the mean, which is given by: $\frac{\sum_{i=1}^{4}\left(x_i - \langle x\rangle\right)^2}{(N-1)^2}$.

While umbrella sampling was performed at $100T_R$ for both the ligand-bound and ligand-free states to explore a broad ensemble of conformations including the unfolded state, for equilibrium simulation runs without umbrellas at various Mg^{2+} concentrations, the temperature was reduced to $95T_R$ to capture only the close to open conformational transition or vice versa. Each trajectory was run for 100 million steps. The equilibration time depends strongly on Mg^{2+} concentration, so simulations at higher Mg^{2+} concentrations were comparatively better converged than at lower Mg^{2+} concentrations.

Supplementary References :

1. Chen J, Poddar NK, Tauzin LJ, Cooper D, Kolomeisky AB, et al. (2014) Single-molecule FRET studies of HIV TAR-DNA hairpin unfolding dynamics. Journal of Physical Chemistry B 118: 12130-12139.
2. Kobitski AY, Nierth A, Helm M, Jaschke A, Nienhaus GU (2007) Mg2+-dependent folding of a Diels-Alderase ribozyme probed by single-molecule FRET analysis. Nucleic Acids Research 35: 2047-2059.
3. Schneidman-Duhovny D, Hammel M, Sali A (2010) FoXS: a web server for rapid computation and fitting of SAXS profiles. Nucleic Acids Research 38: W540-W544.
4. Franke D, Jeffries CM, Svergun DI (2015) Correlation Map, a goodness-of-fit test for one-dimensional X-ray scattering spectra. Nature Methods 12: 419-422.
5. Kumar R, Keyes T (2012) The relation between the structure of the first solvation shell and the IR spectra of aqueous solutions. Journal of Biological Physics 38: 75-83.
6. Noel JK, Whitford PC, Onuchic JN (2012) The shadow map: a general contact definition for capturing the dynamics of biomolecular folding and function. Journal of Physical Chemistry B 116: 8692-8702.
7. Hayes RL, Noel JK, Mandic A, Whitford PC, Sanbonmatsu KY, et al. (2015) Generalized Manning Condensation Model Captures the RNA Ion Atmosphere. Phys Rev Lett 114.
8. Hayes RL, Noel JK, Mohanty U, Whitford PC, Hennelly SP, et al. (2012) Magnesium Fluctuations Modulate RNA Dynamics in the SAM-I Riboswitch. J Am Chem Soc 134: 12043-12053.
9. Hayes Ryan L, Noel Jeffrey K, Whitford Paul C, Mohanty U, Sanbonmatsu Karissa Y, et al. (2014) Reduced Model Captures Mg2+-RNA Interaction Free Energy of Riboswitches. Biophysical Journal 106: 1508-1519.
